# Retraction Note: Overexpressing p130/E2F4 in mesenchymal stem cells facilitates the repair of injured alveolar epithelial cells in LPS-induced ARDS mice

**DOI:** 10.1186/s13287-022-02867-3

**Published:** 2022-05-10

**Authors:** Xiwen Zhang, Jianxiao Chen, Ming Xue, Yuying Tang, Jingyuan Xu, Ling Liu, Yingzi Huang, Yi Yang, Haibo Qiu, Fengmei Guo

**Affiliations:** Department of Critical Care Medicine, Zhongda Hospital, School of Medicine, Southeast University, No.87 Dingjiaqiao Road, Gulou District, Nanjing, 210009 People’s Republic of China

## Retraction Note: Zhang et al. Stem Cell Research & Therapy (2019) 10:74 10.1186/s13287-019-1169-1

The Editors-in-Chief have retracted this Article. Concerns were raised about image overlap between Fig. 1 panels c and d (14d samples). The authors provided original data which contained further overlap between images with different labels. The Editors-in-Chief therefore no longer have confidence in the data presented in this article.

Ming Xue, Jingyuan Xu, Yi Yang and Fengmei Guo agree to this retraction. Xiwen Zhang, Jianxiao Chen, Yuying Tang, Ling Liu, Yingzi Huang and Haibo Qiu have not responded to any correspondence from the publisher about this retraction notice.

